# Impact of bilateral surgery on time to treatment in patients with breast cancer undergoing mastectomy – a retrospective cohort study

**DOI:** 10.1186/s12885-025-15389-x

**Published:** 2025-12-19

**Authors:** Martin Heidinger, Sarah Staehelin, Florian S. Halbeisen, Nadia Maggi, Julie M. Loesch, Rama Kiblawi, Marie Louise Frevert, Fabienne D. Schwab, Christian Kurzeder, Giacomo Montagna, Walter P. Weber

**Affiliations:** 1 Breast Clinic, University Hospital Basel, Basel, Switzerland; 2 Gynecology & Obstetrics, University Hospital Basel, Basel, Switzerland; 3 University of Basel, Spitalstrasse 21, Basel, 4051 Switzerland; 4 Surgical Outcome Research Center, University Hospital Basel and University of Basel, Basel, Switzerland; 5 Breast Service, Department of Surgery, Memorial Sloan Kettering Cancer Center, New York, NY USA

**Keywords:** Breast cancer, Bilateral mastectomy, Risk-reducing mastectomy, Adjuvant treatment

## Abstract

**Introduction:**

It is unclear whether patients with breast cancer (BC) who undergo mastectomy experience delays in adjuvant treatment when bilateral surgery is performed. This study was conducted with the objective of addressing this knowledge gap.

**Methods:**

Stage 0-III BC patients who underwent mastectomy (including conventional mastectomy, nipple-sparing mastectomy [NSM], and skin-sparing mastectomy [SSM]) at a Swiss university hospital between 01/2013 and 12/2023 were identified from a prospectively maintained database. Multivariable Cox proportional hazards regression models were used to detect predictors of time to adjuvant treatment.

**Results:**

A total of 394 patients were included, 308 (78.2%) of whom underwent unilateral mastectomy (UM) and 86 (21.8%) of whom underwent bilateral mastectomy (BM). Compared with patients who underwent UM, those who underwent BM were younger (median age [years] 49 vs. 60), more likely to carry a pathogenic germline BRCA variant (28.7% vs. 2.6%), presented with bilateral breast cancer (14.9% vs. 0%), and underwent neoadjuvant chemotherapy (34.5% vs. 12.4%). Patients who underwent BM were more likely to receive NSM or SSM (81.4% vs. 52.9%) and to develop wound healing disorders (20.7% vs. 11.4%). On univariable analysis, no differences in time to any adjuvant treatment were observed between BMs and UMs (median [days] 34 vs. 33; *p* = 0.444). Multivariable analysis suggested that bilateral mastectomy was associated with a shorter time to adjuvant chemotherapy (HR 2.54, 95% CI 1.15–5.58). Short-term postoperative complications were associated with prolonged time to any adjuvant treatment (HR 0.50, 95% CI 0.36–0.71) and time to adjuvant chemotherapy (HR 0.37, 95% CI 0.17–0.79).

**Conclusion:**

Undergoing BM did not result in a delay in time to adjuvant treatment in comparison to UM.

## Introduction

Rates of bilateral mastectomy (BM) in patients with breast cancer (BC) have been increasing in the United States, ranging from 7% to 12.7%. In Europe and Canada, stable to increasing rates between 6.4% and 7.0% have been reported [1–7]. The reasons for this rising demand are multifactorial. Patients with unilateral breast cancer may opt for more extensive surgical interventions to mitigate the risk of contralateral BC, reduce cancer worry, address preoperative abnormal findings detected in the contralateral breast on MRI, enhance survival outcomes, and/or pursue symmetrization objectives [8–15]. Such surgical interventions have been demonstrated to reduce the risk of BC, potentially leading to better survival outcomes for selected patients with pathogenic germline BRCA 1 or 2 variants (gBRCApv) [16, 17], and may be indicated in cases of bilateral breast lesions. However, current evidence does not support the utilization of this approach for other patient cohorts, and there is a concomitant increase in surgical complications [18–20].

The impact of BM on the time to treatment initiation remains inconclusive. While some studies have described a prolonged time to treatment following BM [21], others have not reported such an association [22–24]. Furthermore, the time to surgery has been described as prolonged for patients receiving mastectomy compared with those receiving breast-conserving surgery, as well as for patients receiving BM compared with those receiving unilateral mastectomy (UM) [21, 25]. Moreover, delays in both the time to surgery and the time to adjuvant treatment have been found to be associated with increased mortality [26].

The objective of this single-center retrospective cohort study was to assess the impact of bilateral surgery on the time to treatment in patients with BC who underwent mastectomy.

## Materials and methods

### Study design & data collection

We performed a retrospective observational cohort study of consecutive patients with stage 0-III BC who underwent mastectomy (including conventional mastectomy, skin-sparing mastectomy [SSM] and nipple-sparing mastectomy [NSM]) between January 2013 and December 2023 at the University Hospital of Basel, Switzerland. Participants were identified from a prospectively maintained institutional database. Baseline characteristics, treatment characteristics, and outcome variables were recorded via the online clinical data management system secuTrial©, which adheres to good clinical practice standards and is maintained by our institutional clinical trial unit. After data cleaning, the final dataset for the present analysis was extracted on November 28, 2024.

### Ethical approval

Ethical approval was obtained from the local ethics committee (Ethikkommission Nordwest- und Zentralschweiz, approval number 2016-01525). Written informed consent was obtained from all participants. The study is reported according to the Strengthening the Reporting of Observational Studies in Epidemiology (STROBE) guidelines.

### Covariates

The clinical tumor stage was determined by clinical examination and imaging, including mammography and ultrasound. Postoperative complications were recorded prospectively during routine postoperative follow-up visits one week and one month after surgery. Short-term complications were defined as those occurring ≤ 30 days postoperatively, including wound healing disorders, surgical site infections (SSI), seroma or hematoma, or secondary hemorrhage requiring surgery. Wound healing disorders included delayed wound healing (primary wound healing > 21 days), skin or nipple necrosis, and flap loss. SSI was defined as the need for antibiotic or invasive treatment. Clinically relevant seroma or hematoma was defined as posing an impairment to the patient’s daily activities and/or requiring invasive procedures. The time to any adjuvant treatment was defined as the number of days from definitive oncological breast surgery to the initiation of the first adjuvant treatment (including endocrine therapy, chemotherapy, or radiotherapy). The time to specific adjuvant treatments, such as chemotherapy or radiotherapy in patients not receiving chemotherapy, was calculated from definitive oncological breast surgery to the initiation of the specific treatment. The time to surgery in patients who underwent upfront surgery was calculated from the date of diagnosis to the date of the first tumor-related surgical procedure of the breast. After neoadjuvant chemotherapy (NACT), the time to surgery was calculated from the date of the last chemotherapy administration to the date of the first tumor-related surgical procedure of the breast.

### Endpoints

The primary aim of this study was to assess whether patients who underwent BM experience delays in adjuvant treatment compared with patients who underwent UM. The secondary endpoints included differences in the time to surgery and the frequency of complications after UM versus BM and their impact on the time to adjuvant treatment.

### Statistical analysis

Descriptive statistics were used to summarize the study characteristics. The results are presented as frequencies with percentages for categorical variables and medians with interquartile ranges (IQR) for continuous variables. Categorical variables were compared between groups via Fisher’s exact test, and continuous variables were analyzed via the Wilcoxon rank-sum test. To assess the relationships between surgical laterality (unilateral vs. bilateral), age, gBRCApv status, pathologic tumor stage, pathologic nodal stage, type of mastectomy, short-term surgical complication, and wound healing disorder with time to any adjuvant treatment and adjuvant chemotherapy, multivariable Cox proportional hazards regression models were employed. All the statistical analyses were performed via R (version 4.3.2) [27].

## Results

### Patient and tumor characteristics

**Fig. 1:**
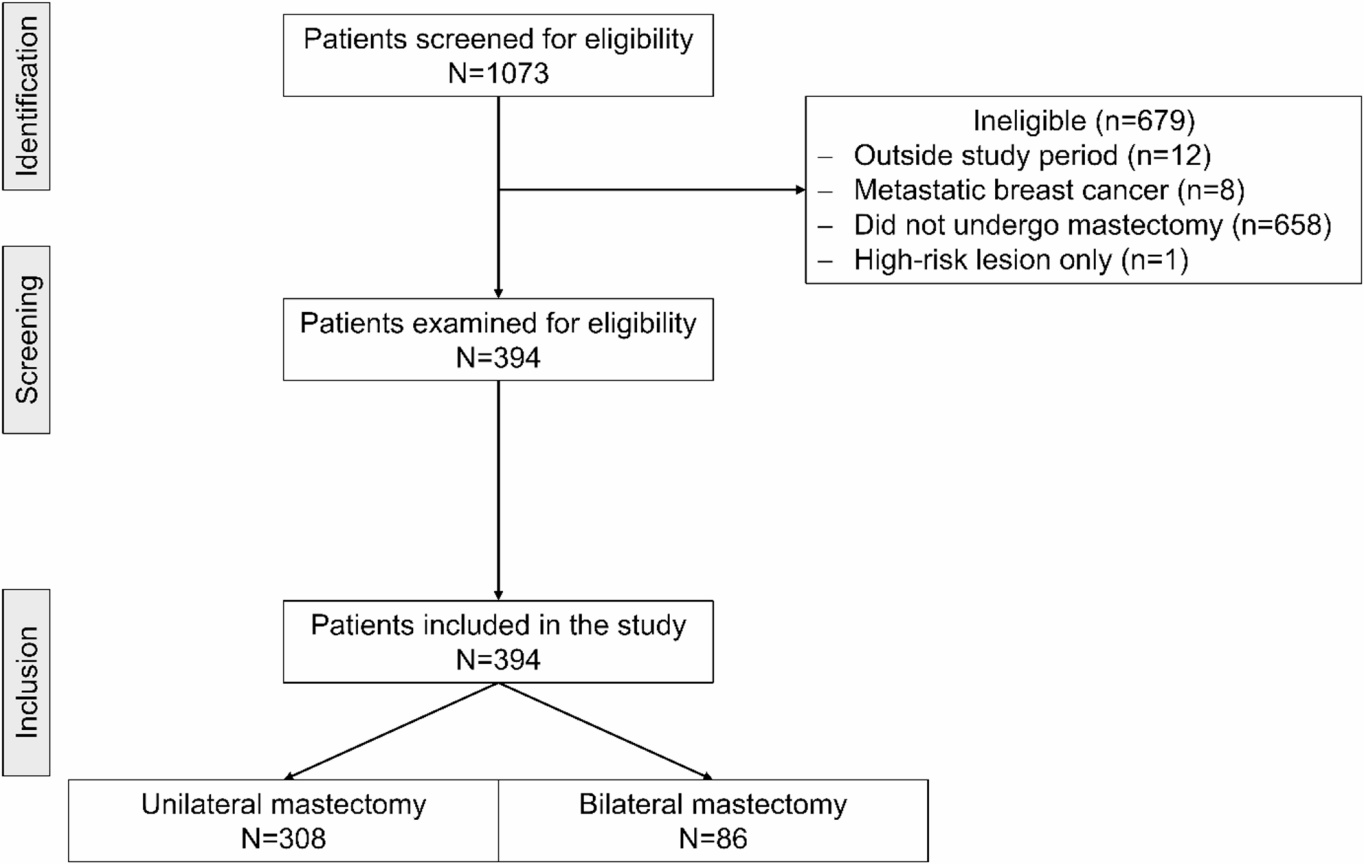
Study flow chart

A total of 394 patients met the eligibility criteria, of whom 78.2% (308/394) underwent UM and 21.8% (86/394) underwent BM (Fig. 1). The median follow-up period was 34.9 months (IQR 15.1–60.4), without differences between the groups (*p* = 0.731). Women who underwent BM were younger (median age [years] 49 vs. 60, *p* < 0.001; Table 1) and more frequently carried a gBRCApv (28.7% vs. 2.6%, *p* < 0.001). Among the patients who underwent BM, 14.9% (13/86) had bilateral BC. While most tumors were hormone receptor (HR)-positive and human epidermal growth factor receptor 2 (Her2)-negative (72.1%, 284/394) and 14.7% (58/394) of patients had Her2-positive BC, the tumors of patients who underwent BM tended to be triple negative (19.5% vs. 9.1%, *p* = 0.063). The clinical tumor and nodal stages did not differ between the groups. However, patients who underwent BM had smaller pathological tumor sizes (pT0/Tis/T1 75.6% vs. 56.5%, *p* < 0.001) and were more frequently pathologically node negative (68.6% vs. 57.5%, *p* < 0.001).

**Table 1:** Patient, tumor, and treatment characteristics

	Total (*n* = 394)	Unilateral (*n* = 308)	Bilateral (*n* = 86)	*P* value
Age (median [Q1,Q3])	58 [47,72]	60 [48,73.5]	49 [41,60.5]	< 0.001
Ethnicity				
White/Caucasian	383 (97.2%)	300 (97.7%)	83 (95.4%)	0.406
Other	10 (2.5%)	7 (2.3%)	3 (3.5%)	
Missing data	1 (0.3%)	1 (0.3%)		
BMI (median [Q1,Q3])	23.45 [20.8,27.1]	23.4 [20.9,26.6]	24.2 [20.7,27.95]	0.508
Menopausal status				
Premenopausal	136 (34.5%)	88 (28.7%)	48 (55.2%)	< 0.001
Postmenopausal	247 (62.7%)	210 (68.2%)	37 (43.0%)	
Missing data	11 (2.8%)	10 (3.3%)	1 (1.1%)	
Any comorbidities	181 (45.9%)	146 (47.6%)	35 (40.2%)	0.273
Pathogenic germline BRCA variant present	33 (8.4%)	8 (2.6%)	25 (28.7%)	< 0.001
Clinical tumor stage				
Tx	4 (1%)	4 (1.3%)		0.473
T0	1 (0.3%)	1 (0.3%)		
Tis	59 (15%)	47 (15.3%)	12 (13.8%)	
T1	125 (31.7%)	90 (29.2%)	35 (40.7%)	
T2	140 (35.5%)	114 (37.1%)	26 (29.9%)	
T3	47 (11.9%)	36 (11.7%)	11 (12.6%)	
T4	17 (4.3%)	15 (4.9%)	2 (2.3%)	
Missing data	1 (0.3%)	1 (0.3%)		
Multifocality	79 (20.1%)	63 (20.5%)	16 (18.4%)	0.762
Clinical nodal stage				
Nx	9 (2.3%)	8 (2.6%)	1 (1.1%)	0.158
N0	272 (69%)	214 (69.5%)	58 (67.4%)	
N1	92 (23.4%)	73 (23.8%)	19 (21.8%)	
N2	7 (1.8%)	6 (2%)	1 (1.1%)	
N3	14 (3.6%)	7 (2.3%)	7 (8%)	
Tumor grade				
Grade 1	56 (14.2%)	40 (13%)	16 (18.4%)	0.006
Grade 2	194 (49.2%)	166 (53.9%)	28 (32.6%)	
Grade 3	138 (35%)	98 (31.9%)	40 (46%)	
Missing data	6 (1.5%)	4 (1.3%)	2 (2.3%)	
Pathologic tumor stage				
pT0	1 (0.3%)		1 (1.1%)	< 0.001
pTis	48 (12.2%)	37 (12.1%)	11 (12.6%)	
pT1	125 (31.7%)	99 (32.1%)	26 (30.2%)	
pT2	92 (23.4%)	81 (26.4%)	11 (12.6%)	
pT3	37 (9.4%)	36 (11.7%)	1 (1.1%)	
pT4	5 (1.3%)	5 (1.6%)		
ypT0/ypTis	41 (10.4%)	24 (7.8%)	17 (19.5%)	
ypT1	24 (6.1%)	14 (4.6%)	10 (11.5%)	
ypT2	17 (4.3%)	11 (3.6%)	6 (6.9%)	
ypT3	2 (0.5%)		2 (2.3%)	
ypT4	2 (0.5%)	1 (0.3%)	1 (1.1%)	
Pathologic nodal stage				
pNX	19 (4.8%)	18 (5.9%)	1 (1.1%)	< 0.001
pN0	182 (46.2%)	146 (47.4%)	36 (41.9%)	
pN1	69 (17.5%)	60 (19.5%)	9 (10.3%)	
pN2	16 (4.1%)	13 (4.2%)	3 (3.4%)	
pN3	22 (5.6%)	21 (6.8%)	1 (1.1%)	
ypNX	3 (0.8%)	2 (0.7%)	1 (1.1%)	
ypN0	54 (13.7%)	31 (10.1%)	23 (26.4%)	
ypN1	19 (4.8%)	13 (4.2%)	6 (6.9%)	
ypN2	5 (1.3%)	2 (0.7%)	3 (3.4%)	
ypN3	5 (1.3%)	2 (0.7%)	3 (3.4%)	
Type of mastectomy				
Conventional Mastectomy	161 (40.9%)	145 (47.2%)	16 (18.4%)	< 0.001
Skin sparing mastectomy	90 (22.8%)	67 (21.8%)	23 (26.4%)	
Nipple sparing mastectomy	143 (36.3%)	96 (31.2%)	47 (54.7%)	
Type of immediate breast reconstruction				
No reconstruction	157 (39.8%)	140 (45.6%)	17 (19.5%)	< 0.001
Implant-based breast reconstruction	101 (25.6%)	63 (20.5%)	38 (43.7%)	
Autologous breast reconstruction	135 (34.3%)	104 (33.8%)	31 (36.0%)	
Missing data	1 (0.3%)	1 (0.3%)		
Type of axillary surgery				
No axillary surgery	35 (8.9%)	28 (9.1%)	7 (8%)	0.365
Sentinel node biopsy	199 (50.5%)	150 (48.7%)	49 (57.0%)	
Tailored axillary surgery	46 (11.7%)	35 (11.4%)	11 (12.6%)	
Axillary lymph node dissection	114 (28.9%)	95 (30.9%)	19 (21.8%)	

*Q1* Quartile 1, *Q3 * Quartile 3

### Treatment characteristics

Patients who underwent BM more frequently underwent NSM/SSM (81.4% vs. 52.9%, *p* < 0.001; Table 1). The type of immediate breast reconstruction differed between the groups (*p* < 0.001). Patients who underwent BM more frequently underwent reconstruction (80.2% vs. 54.2%), which was most frequently implant-based (43.7% vs. 20.5%). Furthermore, patients who underwent BM more frequently received neoadjuvant chemotherapy (34.5% vs. 12.4%, *p* < 0.001), while adjuvant treatments were similar between the groups (Table 2).

**Table 2:** Neoadjuvant and adjuvant treatment characteristics

	Total (*n* = 394)	Unilateral (*n* = 308)	Bilateral (*n* = 86)	*P* value
Neoadjuvant chemotherapy	68 (17.3%)	38 (12.4%)	30 (34.5%)	< 0.001
Any adjuvant treatment	317 (80.5%)	252 (82.1%)	65 (74.7%)	0.128
Adjuvant chemotherapy	86 (21.8%)	73 (23.8%)	13 (14.9%)	0.105
Adjuvant targeted therapy ^a^	54 (13.7%)	39 (12.7%)	15 (17.2%)	0.291
Adjuvant anti-hormonal therapy	259 (65.7%)	209 (68.1%)	50 (57.5%)	0.074
Adjuvant radiotherapy	139 (35.3%)	106 (34.5%)	33 (37.9%)	0.611

^a^including anti-Her2 therapies, immunotherapy, and PARP inhibitors

### Surgical complications

While no differences in the frequency or severity of surgical complications were observed between the two groups (Table 3), delayed wound healing was more prevalent in patients who underwent BM (20.7% vs. 11.4%, *p* = 0.032).

**Table 3:** Surgery-related morbidity

	Total (*n* = 394)	Unilateral (*n* = 308)	Bilateral (*n* = 86)	*P* value
Any complication	111 (28.2%)	80 (26.1%)	31 (35.6%)	0.105
Clavien‒Dindo classification				
Grade I	37 (9.4%)	27 (8.8%)	10 (11.5%)	0.201
Grade II	9 (2.3%)	8 (2.6%)	1 (1.1%)	
Grade IIIa	7 (1.8%)	6 (2%)	1 (1.1%)	
Grade IIIb	58 (14.7%)	39 (12.7%)	19 (21.8%)	
Surgical site infection	27 (6.9%)	18 (5.9%)	9 (10.3%)	0.258
Any wound healing disorder ^a^	53 (13.5%)	35 (11.4%)	18 (20.7%)	0.032
Seroma or hematoma	63 (16%)	45 (14.7%)	18 (20.7%)	0.185

^a^Delayed wound healing, flap loss, nipple or skin necrosis

### Time to treatment

**Fig. 2:**
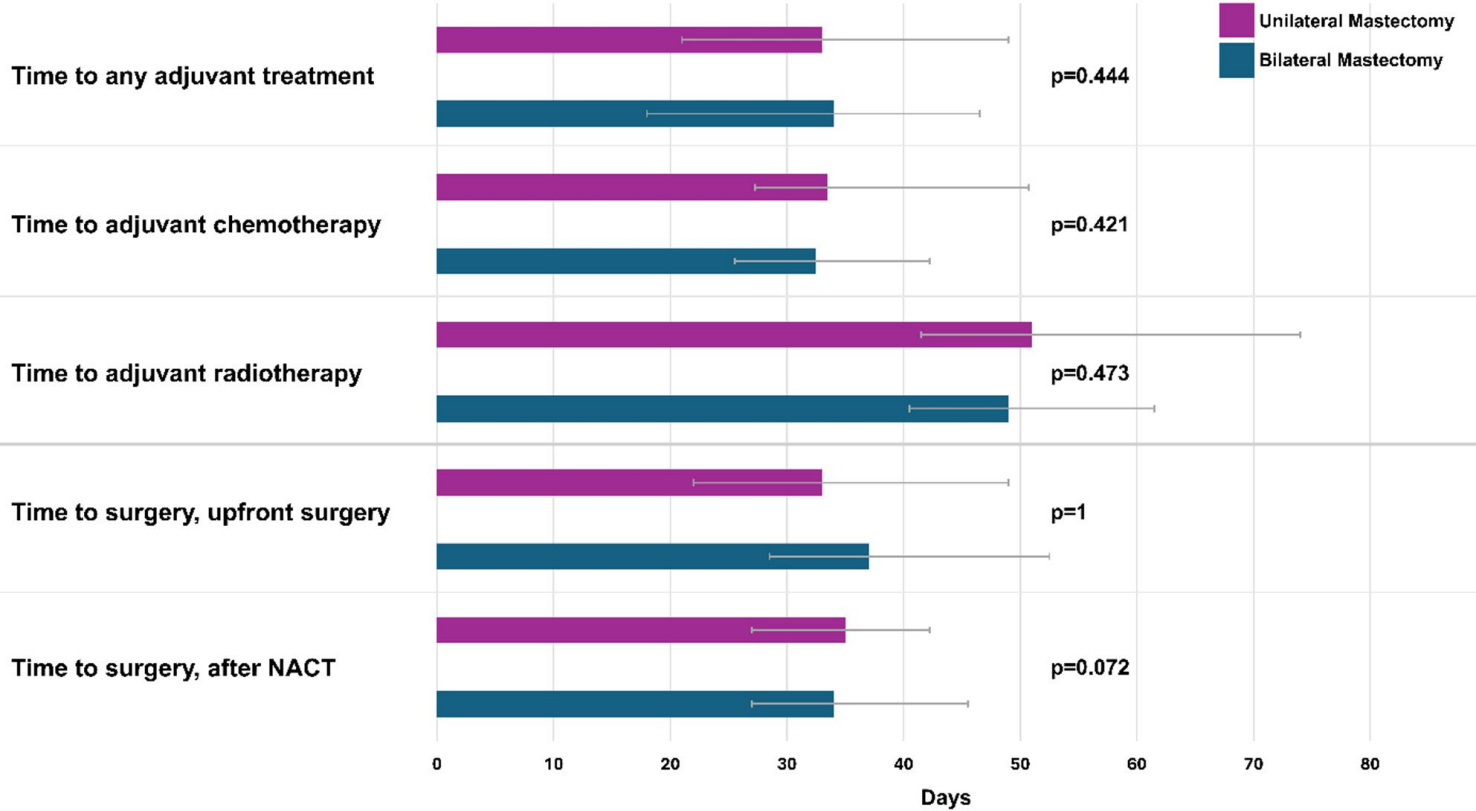
Time to treatment in patients who underwent unilateral or bilateral mastectomy

On univariable analysis, no differences were observed in the time to any adjuvant treatment (median [days] BM: 34, UM: 33, *p* = 0.444; Fig. 2). Furthermore, the time to adjuvant chemotherapy (median [days] BM: 32.5, UM: 33.5, *p* = 0.421) and the time to adjuvant radiotherapy in patients without chemotherapy (median [days] BM: 49, UM: 51, *p* = 0.473) did not differ between the groups. Similarly, no differences were observed in the time to surgery between patients who underwent upfront surgery (median days from diagnosis to BM surgery: 34, UM: 35, *p* = 1) and those who underwent NACT (median days from the last dose of chemotherapy to BM surgery: 37, UM: 33, *p* = 0.072).

**Fig. 3:**
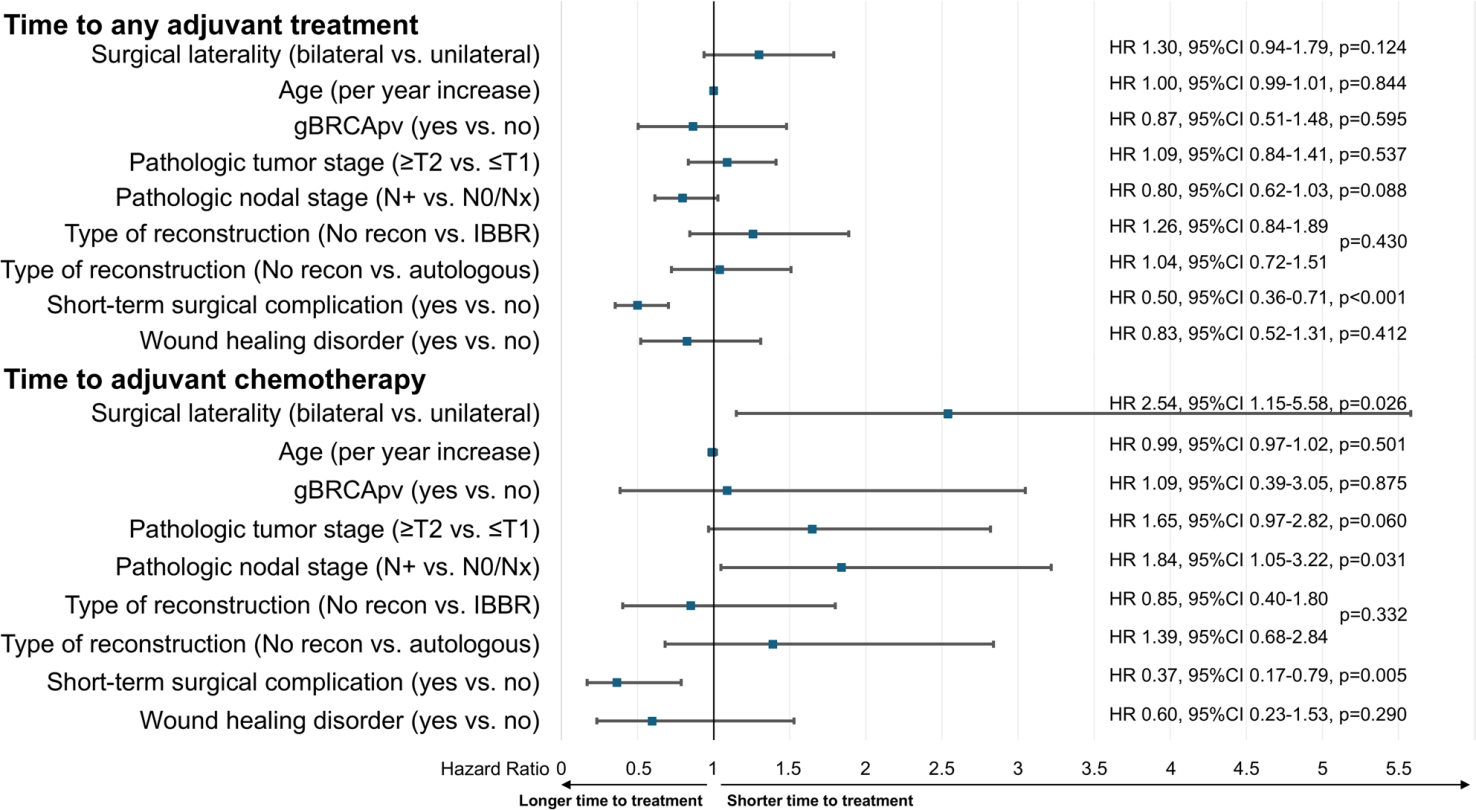
Multivariable analysis of predictors for time to adjuvant treatment. gBRCApv – pathogenic germline BRCA variant; recon – reconstruction; IBBR – implant-based breast reconstruction; HR – hazard ratio

Multivariable analysis suggested that bilateral mastectomy was associated with a shorter time to adjuvant chemotherapy (HR 2.54, 95% CI 1.15–5.58; *p* = 0.026; Fig. 3). The only factors associated with a longer time to treatment were surgery-related complications, both for any adjuvant treatment (HR 0.50, 95% CI 0.36–0.71, *p* < 0.001) and for adjuvant chemotherapy (HR 0.37, 95% CI 0.17–0.79, *p* = 0.005). Pathological node-positivity was associated with a shorter time to adjuvant chemotherapy (HR 1.84, 95% CI 1.05–3.22, *p* = 0.031).

## Discussion

Despite the existence of evidence that BM does not improve oncological outcomes, albeit in a small group of patients, increases complication rates, and does not improve patient-reported outcomes (PROs), the use of BM has increased markedly over the last two decades [1–3, 7, 18–20]. Especially the potential increase of surgery-related complications has raised concerns, as these can impact the initiation of adjuvant treatments and, consequently, survival outcomes. Conflicting results concerning the delay in treatment in patients with BM have been reported. The present study sought to address this knowledge gap by comparing patients who underwent UM or BM at a tertiary cancer center over a 10-year period. We found that BM was not associated with a delay in treatment initiation.

The time to treatment in this study was comparable to that in previously published results, which were mainly from the United States. The time to any adjuvant therapy was 33 days in our study, with previous studies reporting durations ranging between 40 and 69 days [21, 22, 24]. Compared with UM, bilateral mastectomy did not delay the time to adjuvant therapy. These findings are consistent with those of previous studies [22, 23]. A study by Sharpe et al.., which analyzed 390’172 patients in the U.S. National Cancer Database (NCDB) between 2003 and 2010, reported delays in adjuvant therapy for patients who underwent BM. However, it should be noted that the study exclusively included patients in the upfront surgery setting, of whom two-thirds underwent conventional mastectomy, and the absolute difference in delay was three days (median days UM: 66; BM 69), which is not clinically relevant [21]. In the present multivariable analysis, no delay but even an association with faster adjuvant chemotherapy after BM was observed. Furthermore, patients with pathological node-positivity were found to have a shorter time to adjuvant chemotherapy initiation. This observation may be attributed to the perceived greater risk-associated treatment pressure in these patients. Patients having undergone BM in the present cohort presented a greater prevalence of gBRCApv, a greater incidence of TNBC, and a greater frequency of NACT. Neoadjuvant chemotherapy shows increasing indications for patients with breast cancer, improving both recurrence-free and overall survival, and allowing for post-neoadjuvant response-driven treatment decisions [28–31]. Patients undergoing NACT typically have a longer lead time to surgery, allowing for a longer period of surgical planning and coordination of surgical logistics. Therefore, no delay in time to surgery was expected. On the other hand, while the association of NACT on surgical complications in patients with contralateral risk-reducing mastectomies and immediate reconstruction was previously shown by Sharpe et al. [21], the potential impact of NACT on time to treatment delays was not yet investigated. Therefore, we sought to address this knowledge gap by including this clinically increasingly important group of patients.

No differences in the time to surgery were observed between patients who underwent upfront surgery and those who underwent NACT, with median durations of 34 days and 34.5 days, respectively. These findings contrast with those of the study by Sharpe et al.., in which patients in the upfront surgery setting who underwent UM had a median time to surgery of 33 days, whereas those who underwent BM were operated on a median of 40 days after diagnosis [21]. Prakash et al.. analyzed data from the U.S. NCDB for patients diagnosed with BC between 2004 and 2015 and reported a longer time to surgery in patients who underwent mastectomy than in those who underwent breast-conserving surgery; however, no clinically relevant differences were observed in the time to surgery between patients who underwent UM or BM, both in the neoadjuvant and upfront settings [25]. In our multidisciplinary breast clinic, patients newly diagnosed with BC have the opportunity to consult with both a breast surgeon and a plastic surgeon during their initial consultation. Additionally, genetic consultations are integrated within the breast clinic. Previous studies have revealed that the logistic challenges associated with surgical planning involving immediate reconstruction are associated with delays in surgery [25, 32]. Moreover, high-volume academic institutions have been found to be associated with the longest time intervals to surgery [25, 33]. The concept in our center appears to ensure an efficient surgical planning process, even in patients planning to undergo bilateral surgery with immediate reconstruction. The highly specialized collaboration with colleagues from plastic surgery is also underscored by the fact that we did not find the type of breast reconstruction to influence the time to adjuvant treatment. This finding is in accordance with those of previous studies, which did not find that breast reconstruction impacts the time to adjuvant treatment [22]. Moreover, prior studies have identified an association between high case volume and reduced complication rates in autologous reconstruction procedures, which were performed in one-third of the present cohort [34, 35]. This observation is particularly noteworthy in light of earlier research indicating that mastectomy with immediate autologous reconstruction results in delays in adjuvant chemotherapy, which has been associated with poorer survival outcomes [36, 37]. In our setting, neither a delay to any adjuvant treatment, nor to adjuvant chemotherapy was observed after autologous reconstruction.

The study revealed that 28.2% of patients experienced complications, with more than half of those (52.3%) necessitating readmission and surgical intervention under general anesthesia. A comparable cohort study by Eck et al.. reported a 26.7% complication rate, with 39% of patients requiring reoperation [22]. A higher incidence of wound healing disorders was observed in patients with BM. Furthermore, these patients undergo immediate breast reconstruction more frequently, a procedure known to increase the risk of postsurgical complications, than conventional mastectomy does [38]. Interestingly, in the present study, these differences in postsurgical morbidity did not persist compared with the number of operated breasts. Patients who underwent BM demonstrated a rate of wound healing disorders of 10.5% (18/172), which was similar to that of patients who underwent UM (11.4%). This finding indicates that surgery-related complications can delay adjuvant treatments, regardless of whether patients undergo UM or BM.

In summary, the time to treatment did not differ between patients who underwent UM or BM in this analysis at a single academic center. While these results are reassuring concerning the individual patient paths within a complex diagnostic and pre- as well as postoperative trajectory, they inform us that the decision to conduct a BM should be based on well-defined medical indications. Bilateral mastectomy remains a potential mutilating surgery that offers no oncological benefits for many patients. However, the risk of complications remains elevated, and no improvement in PROs has been reported. It is imperative to acknowledge the pivotal role that surgeon recommendations play in the context of BM. Surgeon recommendation against BM, in the absence of clear indications, has been demonstrated to result in a reduction of BM rates [18, 20]. Importantly, decision to perform a BM should incorporate a risk assessment for postoperative complications, and the planned treatment should aim to reduce these complications as much as possible, as they were found to delay adjuvant treatment, which could negatively affect oncological outcomes [26]. Furthermore, future research should address whether delays in treatment impact PROs, which is becoming an increasingly important endpoint in survivorship research of patients with BC.

### Limitations

The limitations of the present study include its retrospective nature and single-center setting, which renders the results susceptible to selection bias and limits their generalizability. As such, the presented results may differ in settings with higher case loads, constrained operating room capacities, and/or limited access to plastic surgery services. Additionally, the study’s limited sample size may preclude the detection of subtle differences.

## Conclusion

The results of this study suggest that BM did not impact the time to treatment initiation. However, surgical complications were found to delay adjuvant treatment, which emphasizes the importance of efforts to perform BM exclusively in patients with evident benefit and to minimize surgical morbidity.

## Data Availability

The data that support the findings of this study are available from the corresponding author upon reasonable request.
